# Posterior cortical atrophy: an investigation of scan paths generated during face matching tasks

**DOI:** 10.3389/fnhum.2013.00309

**Published:** 2013-06-28

**Authors:** Benjamin P. Meek, Keri Locheed, Jane M. Lawrence-Dewar, Paul Shelton, Jonathan J. Marotta

**Affiliations:** ^1^Perception and Action Laboratory, Department of Psychology, University of ManitobaWinnipeg, MB, Canada; ^2^Department of Internal Medicine, Section of Neurology, University of ManitobaWinnipeg, MB, Canada

**Keywords:** posterior cortical atrophy, eye movements, scan paths, face perception, vision disorders

## Abstract

When viewing a face, healthy individuals focus more on the area containing the eyes and upper nose in order to retrieve important featural and configural information. In contrast, individuals with face blindness (prosopagnosia) tend to direct fixations toward individual facial features—particularly the mouth. Presented here is an examination of face perception deficits in individuals with Posterior Cortical Atrophy (PCA). PCA is a rare progressive neurodegenerative disorder that is characterized by atrophy in occipito-parietal and occipito-temporal cortices. PCA primarily affects higher visual processing, while memory, reasoning, and insight remain relatively intact. A common symptom of PCA is a decreased effective field of vision caused by the inability to “see the whole picture.” Individuals with PCA and healthy control participants completed a same/different discrimination task in which images of faces were presented as cue-target pairs. Eye-tracking equipment and a novel computer-based perceptual task—the *Viewing Window* paradigm—were used to investigate scan patterns when faces were presented in open view or through a restricted-view, respectively. In contrast to previous prosopagnosia research, individuals with PCA each produced unique scan paths that focused on non-diagnostically useful locations. This focus on non-diagnostically useful locations was also present when using a restricted viewing aperture, suggesting that individuals with PCA have difficulty processing the face at either the featural or configural level. In fact, it appears that the decreased effective field of view in PCA patients is so severe that it results in an extreme dependence on local processing, such that a feature-based approach is not even possible.

## Introduction

Imagine looking through a family photo album, and realizing that you do not recognize the people in the photographs, or finding that the walls in your home sometimes change color when you blink your eyes. These are some of the issues that RB, a 77-year-old female, began experiencing more than 5 years ago, despite an absence of neurological injury such as stroke or head injury. Preliminary investigations suggested that RB has prominent deficits in recognizing faces and line drawings of objects, along with significant problems seeing multiple objects in an array and a deficit in global processing (e.g., when shown a photo of a camera, RB mistook the lens of the camera for a tunnel). RB also reported experiencing color “hallucinations.” Despite these perceptual impairments, RB did not show any significant deficits in memory or reasoning. This unusual pattern of deficits illustrates a rare neurodegenerative disorder called Posterior Cortical Atrophy (PCA).

PCA, also referred to as Benson's disease (Benson et al., 1988) or the visual variant of Alzheimer's disease (AD; Bokde et al., 2001; Boxer et al., 2003) is a progressive neurodegenerative disorder that is associated with significant impairments in higher visual processing, while at early stages, memory, reasoning, and insight remain relatively intact (Chan et al., 2001; Charles and Hillis, 2005; Crutch and Warrington, 2007). The initial symptoms of PCA often include problems such as achromatopsia, prosopagnosia, object agnosia, environmental agnosia, alexia, agraphia, left-right disorientation, optic ataxia, oculomotor apraxia, dressing apraxia, visual neglect, and simultanagnosia (Chan et al., 2001; Charles and Hillis, 2005; Crutch and Warrington, 2007; Mendez et al., 2007; Giovagnoli et al., 2009). At later stages, individuals with PCA show impairments in memory, learning, language, and reasoning, and may appear similar to typical AD.

PCA is characterized by progressive bilateral atrophy in the posterior areas of the brain (e.g., occipito-parietal and occipito-temporal areas), often with a predominance in the right hemisphere (Kaida et al., 1998; Goethals and Santens, 2001; Nestor et al., 2003; Caine, 2004; Charles and Hillis, 2005; Kirshner and Lavin, 2006; McMonagle et al., 2006; Mendez et al., 2007; Whitwell et al., 2007). Magnetic resonance imaging (MRI) investigations of PCA show evidence of atrophy in the middle temporal area of the inferior occipito-temporal junction (Caine, 2004), right fusiform gyrus, parahippocampal cortex (Joubert et al., 2003), occipital poles (Chan et al., 2001), right occipital gyrus (Boxer et al., 2003), Brodman's areas 17, 18, and 19, area 7 b and 7 m of the posterior parietal cortex, and Brodman's area 23 of the posterior cingulate cortex (Caine, 2004). In addition, research with single-photon emission computed tomography (SPECT) and positron emission tomography (PET) have shown decreased blood flow and reduced blood-glucose metabolism, bilaterally, in occipito-parietal and occipito-temporal cortices (Bokde et al., 2001; Goethals and Santens, 2001; Mendez, 2001; Boxer et al., 2003; Giovagnoli et al., 2009) which includes the lateral occipital cortex (Giovagnoli et al., 2009), primary visual cortex (V1), and sometimes the frontal eye fields (Bokde et al., 2001; Nestor et al., 2003).

PCA patients often demonstrate impairments in global processing or the ability to view the visual world as a coherent whole. Traditionally, this deficit in PCA has been labeled as simultanagnosia. Simultanagnosia, results in an inability to perceive more than a single object or object component at a time (Luria, 1959; Rizzo and Vecera, 2002; Moreaud, 2003; Montoro et al., 2011). Simultanagnosia is also a component of Balint's syndrome and has been linked to other visual impairments such as optic ataxia, gaze apraxia, agraphia, and problems navigating the environment (Farah et al., 1998; Duncan et al., 2003). Although a key characteristic of simultanagnosia is a selective deficit in the identification of global forms, eye tracking studies have shown scan paths that closely trace the shape of these misidentified global forms (Clavagnier et al., 2006; Dalrymple et al., 2007, 2009, 2010). Recently, however, Crutch (2013) has cautioned that one must be careful diagnosing simultanagnosia in PCA, as the effective field of vision can be limited without overt simultanagnosia, as observed in natural aging and in patients with right parietal lobe lesions (Russell et al., 2012).

When viewing faces, healthy individuals tend to look at the region of the face containing the eyes and upper-nose, since it provides important featural and configural information that allows for rapid and accurate face-identification (Rossion et al., 2000; Lobmaier et al., 2008; Chaby et al., 2011). Indeed, eyes have been found to be a primary oculomotor target regardless of their location (Levy et al., 2013). In contrast, individuals with prosopagnosia, a disorder impairing the ability to recognize a face, spend more time looking at the lower regions of a face (e.g., mouth), external features (e.g., hairline, jawline, outer area of cheeks), or individual features compared to healthy individuals (Le et al., 2003; Caldara et al., 2005; Barton et al., 2006; Bukach et al., 2008; Orban De Xivry et al., 2008). Some researchers have suggested that prosopagnosia is the result of a deficit in holistic/configural representations of faces, and that these individuals are forced to rely solely on featural representations (Le et al., 2003; Caldara et al., 2005; Barton et al., 2006; Bukach et al., 2008; Orban De Xivry et al., 2008). This type of parts-based strategy could explain the observed differences in scan patterns between healthy individuals and those with prosopagnosia, and reflect a deficit in configural processing, which may result in an over reliance on featural representations, among individuals with prosopagnosia.

The present study sought to determine which areas of the face individuals with PCA spend the most time fixated on. An understanding of which regions attract individuals with PCA may reveal more about the nature of their deficits in face recognition and suggest the underlying cause for these impairments. To meet these goals, a face-matching task was completed across two experiments. The first experiment utilized eye tracking during an open-view task, since previous research has used this technology to examine visual scan paths that are associated with face perception. The second experiment, involved a restricted-view task, the newly developed Viewing Window task (Baugh and Marotta, 2007, 2009; Baugh et al., 2011; Lawrence-Dewar et al., 2012), in which only a small portion of an image is clearly visible at one time. Restricting vision is thought to encourage a serial, or part-by-part processing strategy, which may have different effects on scan paths in PCA and healthy populations.

## Materials and methods

The methods common to both experiments will be described first, followed by detailed information that is specific to each experiment.

### Statement on ethics

All procedures were reviewed and approved by the Human Research Ethics Board at the University of Manitoba. In addition, approval of MRI studies were also reviewed and approved by the Human Research Ethics Board for the National Research Council—Institute for Biodiagnostics. Prior to enrolment, all participants provided written informed consent.

### Participants

Experiment 1 examined the performance of patients with PCA, aged healthy individuals, and a group of healthy undergraduate students in an open-view face matching task. Experiment 2 examined the performance of these individuals during a restricted view face matching task. All PCA patients had normal or corrected-to-normal visual acuity, as determined by either their neuro-opthalmologist or optometrist. Healthy individuals were pre-screened for normal or corrected-to-normal visual acuity.

Prior to testing, PCA patients and aged healthy individuals completed several behavioral tests to assess cognitive function including: a famous faces task, the short form Benton Face Recognition Task (FRT), the short form Boston Naming Task, a finger-tapping task (FTT), a phonemic verbal fluency task, or controlled oral word association task (COWAT), the Mini Mental State Exam (MMSE), and the Dementia Rating Scale II (DRS-II). A simple computer-based, reaction time task was completed in which participants pressed a spacebar when a target was presented on the screen. In-house developed object identification and shape-matching tasks were also administered. The object identification task consisted of 18 color images of single objects (e.g., lamp) presented on a computer screen. The computer-based shape-matching task was developed to determine if PCA patients could match serially presented objects after a brief delay. The objects were white computer generated rectangles, based on Blake shapes (Blake, 1992), presented on a black background. The scores of these tests and demographic information of each of the patients and age-matched controls are listed in Table 1.

**Table 1:** **Demographic information and behavioral scores of five patients with PCA and aged-healthy control participants**.

		**RB**	**SS**	**AP**	**MTB**	**PLH**	**C1**	**C2**	**C3**	**C4**	**C5**
Age		76	66	78	67	62	63	67	80	74	75
Sex		F	M	F	F	F	F	F	F	F	M
Handedness		R	R	L	R	R	R	R	R	R	R
Benton FRT		<37 SI	<37 SI	47 Norm	<37 SI	<37 SI	51 Norm	51 Norm	52 Norm	54 Norm	54 Norm
Famous faces (%)		2	44	50	58	12	92	66	58	60	62
Object ID (%)		27.8	72.2	100	100	72.2	100	100	100	94.4	94.4
Object count (%)		85.7	42.9	78.6	64.3	28.6	100	100	100	92.9	100
Shape-match (%)		83.0	100.0	100.0	94.4	N/A	100	N/A	97.2	97.2	97.2
Boston naming (%)		20.0	46.7	100.0	73.0	20.0	100	73.0	100	100	93.3
Finger tap right (Hz)		1.9	2.6	1.2	1.5	2.8	3.6	3.1	3.0	3.0	5.0
Finger tap left (Hz)		1.9	1.9	1.4	1.2	2.8	3.6	2.7	2.5	3.1	3.6
Median reaction time (ms)		559.5	717.0	641.5	672.0	N/A	286.0	N/A	234.0	375.5	301.0
Phonemic fluency (total words)	F	17	19	16	12	N/A	8	19	13	17	18
	A	10	14	12	15	N/A	4	14	14	13	15
	S	16	20	17	16	N/A	11	14	15	12	13
MMSE		26	28	28	28	24	30	30	30	30	30
DRS-2 score		126	129	134	125	108	142	136	135	139	142
Percentile		6–10	6–10	29–40	3–5	<1st	82–89	29–40	29–40	60–71	82–89

Abbreviations: FRT, Benton Facial Recognition Test; SI, Severely Impaired; MMSE, Mini-mental state examination; DRS-2, Dementia Rating Scale.

**Figure 1:**
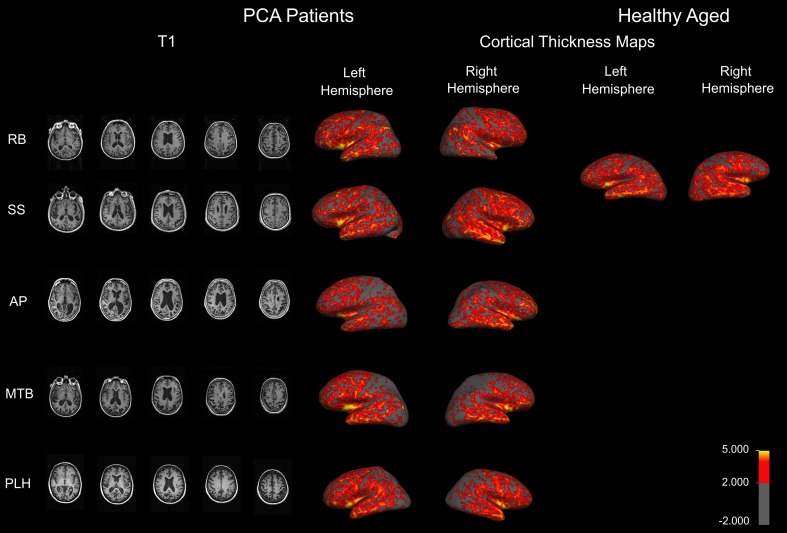
**Anatomical MRIs revealing atrophy in PCA patients.** Sample axial slices from the high-resolution T1 weighed image. Cortical thickness maps obtained in the five patients with PCA and a healthy aged control. Maps are overlaid on inflated brains, so as to display thickness of cortex in sulci. A color spectrum is applied to indicate measured thickness (in mm) indicated on the scale bar.

To examine the extent and distribution of atrophy in all of the PCA patients, an MRI was performed at the National Research Council—Institute for Biodiagnostics (Winnipeg, MB, Canada) using a 3 T Tim Trio MRI system (Siemens, Erlangen, Germany) with integrated head coil. The same scans were also acquired for an additional aged healthy control as part of another study. A high resolution, T1 weighted anatomical image was acquired using a MPRAGE sequence (256 × 256 matrix, in-plane resolution 1 × 1 mm, 176 slices, slice thickness 1 mm, *TR* = 1900 ms, *TE* = 2.2 ms). Five representative images from the T1 are shown in Figure 1 for each patient. A slice was first selected in which the anterior commissure was visible then, moving in a dorsal direction, the 10th slice was selected four more times.

Anatomical data from each patient were analyzed individually to measure cortical thickness using Freesurfer v4.5 (MGH, USA, http://surfer.nmr.mgh.harvard.edu/). The followed workflow includes motion correction, skull stripping, automated Talairach transformation, white and gray matter segmentation, and intensity normalization. Further technical details of the automated procedures have been described (Dale et al., 1999; Fischl et al., 1999a,b, 2001, 2002, 2004a,b; Fischl and Dale, 2000; Segonne et al., 2004; Jovicich et al., 2006; Han et al., 2006). Thickness maps were rendered onto an inflated brain template to reveal measurements of cortex in the sulci. A color spectrum was applied with yellow (5 mm) and red (greater than 2 mm) indicating areas of thicker cortex and gray indicating thin cortex (less than 2 mm).

#### Patient information

Individuals diagnosed with PCA by a local neurologist (P.S.) were informed of the opportunity to participate in the present research study.

***Patient RB***. RB initially experienced difficulty recognizing faces and reported changes in color perception. At the time of testing, RB had impairments in face recognition, object perception, shape matching, figure copying, color identification, reading, writing, and visual search. There was no evidence of hemispatial neglect or spatial disorientation, and normal scores on executive functioning tests that did not involve visual stimuli. RB showed a tendency to replicate one specific detail and an inability to connect details into a coherent whole during figure copying, and focused on single features during object identification, indicating simultanagnosia. Though RB suffers from early macular degeneration and mild cataracts, these issues do not adversely affect her visual acuity. An MRI revealed atrophy in RB's right anterior temporal lobe (Figure 1).

***Patient SS***. SS began experiencing problems navigating familiar environments, finding objects in plain view, and managing his family's finances. A neurologist assessment revealed that SS shows signs of optic ataxia, ideomotor apraxia, and constructional apraxia, in misreaching to visual targets, difficulty imitating hand movements, and poor performance on motor sequencing tasks. He also demonstrates difficulties reading, writing, recognizing objects, spatial orientation, and mild memory impairments. SS's visual object agnosia seems to be a result of a reduced effective field of vision, as he will often identify only a single component of a target object, and his figure copying consists of disconnected, isolated object features. He also fails to identify objects presented in his lower left and right hemifields, indicating impaired spatial attention, and suffers from mild macular degeneration. An MRI revealed mild diffuse atrophy and enlargement of the ventricles (Figure 1).

***Patient AP***. AP reports difficulty reading, stating that the words “jump” on her, and she struggles to find her place in a line of text. However, AP's reading difficulties improve when a restricted window is used to allow letter-by-letter reading. When lines of print were isolated on a page, sentence reading was similarly found to be normal. Her vision fatigues, such that it is increasingly difficult to keep on line or to draw. Her drawing errors were characterized by difficulty relating local features to the overall shape, such as placing the stem on a flower. AP scored normally on tests of face perception and object recognition. She remained non-aphasic and there was no anomia. She demonstrated misreaching consistent with optic ataxia. The later course was characterized by progressive right, more than left, hemispatial neglect, right tactile extinction, dressing apraxia, right hand apraxia and alien hand, and progressive asymmetrical rigidity, bradykinesia and Parkinsonian gait, consistent with Corticobasal Syndrome. A single photon emission computed tomography (SPECT) scan demonstrated moderate hypoperfusion of the left occipito-parietal area and mild changes in the right posterior parietal area. An MRI indicates atrophy in similar areas (Figure 1).

***Patient MTB***. MTB is affected by predominant problems with motor coordination, such as agraphia, unsteady gait, and dressing apraxia. She also showed evidence of left/right confusion, left hemispatial neglect, and spatial and environmental disorientation. MTB demonstrated difficulty reading full words that improved when using a restricted window to view a single letter at a time. MTB's neurologist reported significant constructional problems when she was asked to copy simple figures, but only minor problems in face and object recognition. An MRI revealed that MTB has atrophy in occipito-parietal areas, as well as some occipito-temporal atrophy (Figure 1).

***Patient PLH***. PLH initially experienced perceptual difficulties and blurred vision. She was referred to a neurologist who noted impairments in object, face, and word recognition that could not be explained by visual dysfunction as well as environmental disorientation and mild apraxia. She proved unable to copy simple line drawings or locate items in plain view. PLH exhibits fragmented visual processing and describes only a single component of an object or individual features without regard to the overall image during recognition tasks. PLH demonstrated some evidence of memory impairment, and showed visual field dysfunction in all four quadrants. Specifically, she experienced left visual extinction in the upper and lower quadrants, and her saccades were slow to initiate both vertically and horizontally. An MRI revealed diffuse cortical atrophy involving both the dorsal and ventral streams (Figure 1).

### Healthy controls

Healthy age-matched controls were recruited from the community. Participants were screened for history of neurological disease or injury and completed tests of cognitive and perceptual function (Table 1). Four aged-matched controls were initially recruited however, following Experiment 1, one participant withdrew from the study voluntarily. Therefore, a fifth age-matched control was recruited to complete Experiment 2.

Twelve young healthy individuals (8 females, 10 right-handed, Mean age = 23.5 years old) were recruited for Experiments 1 and 2 from the University of Manitoba's Introduction to Psychology Subject Pool, and received course credit for their participation. Participants were right-handed, fluent in English, and had normal or corrected-to-normal vision.

## Methodology

### Experiment 1—face perception in an open-view task

#### Participants

Three PCA patients (RB, SS, AP) and four age-matched healthy participants (4 females, right-handed, Mean age = 71 years old) participated in Experiment 1, along with all of the younger control participants. Two of the PCA patients, (MTB and PLH), were unable to participate in Experiment 1 due to problems in maintaining fixation long enough for the eye-tracker to calibrate.

#### Procedure

Prior to the start of the experiment, all participants received written and verbal instructions as well as a demonstration of the eye-tracking equipment used in the study. Participants were seated at a table and positioned with their head resting on a chin rest ~50 cm from a 20.1" LCD monitor running at a resolution of 1600 × 1200 and at 60 Hz. An Eye-link II (250 Hz sampling rate, spatial resolution <0.5°; SR Research Ltd., Osgoode, ON, Canada) was used to record eye-movements throughout the experiment. Each participant was first calibrated using a nine-point calibration screen and validated to less than 1° of error. Pupil and CR-based tracking were used in monocular mode, and participants were tested individually at a station consisting of a 3.2 GHz computer, keyboard, and monitor.

Each experimental trial, presented by in-house software written in Matlab® 2008a (MathWorks, Natick, MA), began with a fixation point that was used for *in vivo* drift correction, to compensate for headband slippage or other small movements in the head-mounted eye-tracker. Following the fixation marker, a “cue” face was presented at the center of the computer screen. Participants were instructed to look at the face until they memorized the image. There were no time limits. The participants then pressed a spacebar when they were finished viewing the “cue” face, at which point a checker-pattern mask appeared for 250 ms. Following the mask, a “target” face then appeared. Cue and target stimuli consisted of 76 oval masked faces (300 pixels across the center; 38 “cue” faces, 38 “target” faces) derived from the Productive Aging Lab database (Minear and Park, 2004), converted to grayscale on a black background. The mask was applied to remove external features such as hair, jewelry, and jaw line. Half of these cue-target pairs were comprised of the same face (matched pair), while half were comprised of two different faces (unmatched pair) with the stimulus presentation order randomized for each participant and at least four trials used as practice at the beginning of the experiment.

The participants were instructed to press the spacebar as soon as they made a same/different decision about the serially presented “cue” and “target” faces, and then required to press the “1” key for “same” and the “2” key for “different.” Due to the difficulty experienced pressing the response buttons within the PCA group, the patients were asked to simply press the spacebar key once they made their decision for the target, and to then lift their left hand slightly from the desk for a “same” response, and to leave their left hand on the table top to respond “different.” Verbal responses could not be used due to the chin rest. All of the patients completed enough training in this response procedure to demonstrate adequate understanding to the experimenter before the experimental trials began. For the PCA group, the experimenter pressed “1” for same, and “2” for different, using a response pad that was connected to the computer keyboard. Healthy participants used the standard key presses for same/different responses. The response key presses terminated the response screen, and initiated the next trial. No feedback was given to the participant with regard to accuracy. The accuracy, and viewing time of the cue and target faces was recorded by the same in-house software and exported to a text file.

#### Data analysis

**Figure 2:**
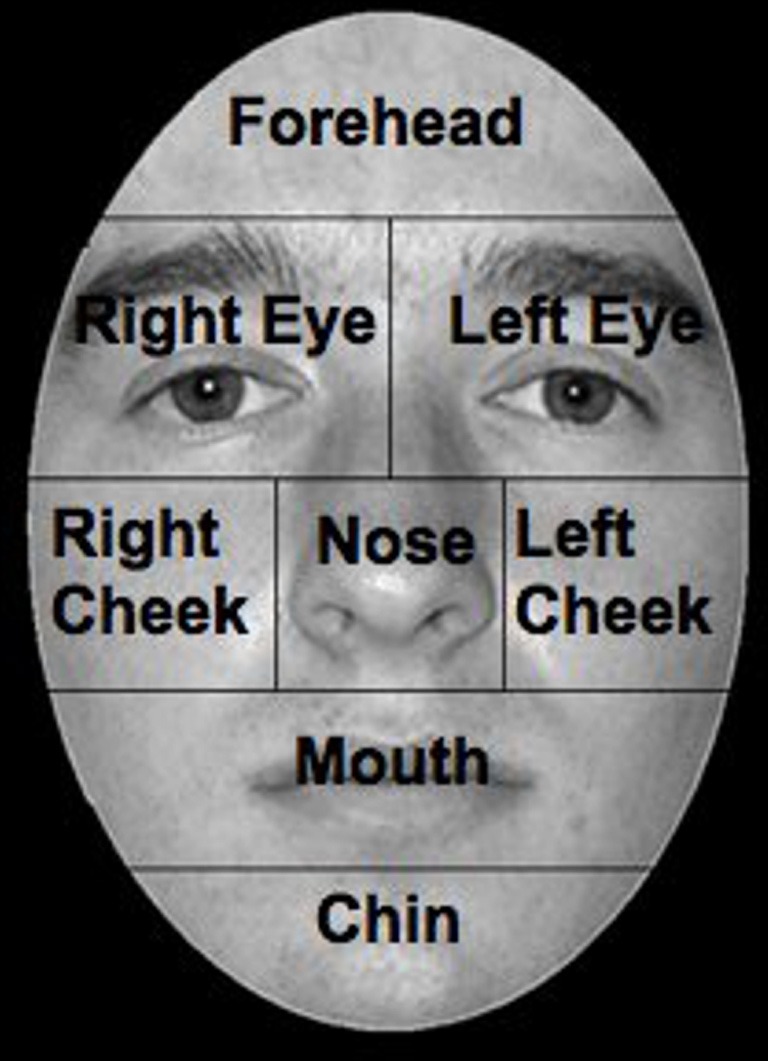
**Facial ROIs, ROIs were defined as areas around anatomical features.** The face presented is a sample of the ones used in the experiments.

Comparisons of the number of errors between control groups were conducted using a two-tailed independent-samples *t*-test, with all results significant at *p* < 0.05. To examine the pattern of eye scanning behavior, faces were divided into eight regions of interest (ROI): left eye, right eye, nose, mouth, chin, forehead, left cheek, and right cheek (Figure 2). ROIs were based on the stimulus; therefore the left eye of the stimulus fell in the right visual field of the participant.

To control for differences in the time spent viewing stimuli, the proportion of fixation duration was calculated by dividing the totals found for each ROI, by the total calculated for the whole stimulus image. These numbers were then averaged for each participant, across stimuli. For the control groups, participant data was pooled for a grand mean of proportion of fixation duration, while means for the PCA group were calculated across stimuli only. A series of paired-samples *t*-tests, corrected for multiple comparisons (*p* < 0.0021) using the Bonferroni procedure, were conducted within each control group to determine if there were any differences in scanning between ROIs. Ninety-five percent confidence limits were then obtained from the control mean proportion of fixation duration using SPSS for comparisons between controls and each individual with PCA.

**Figure 3:**
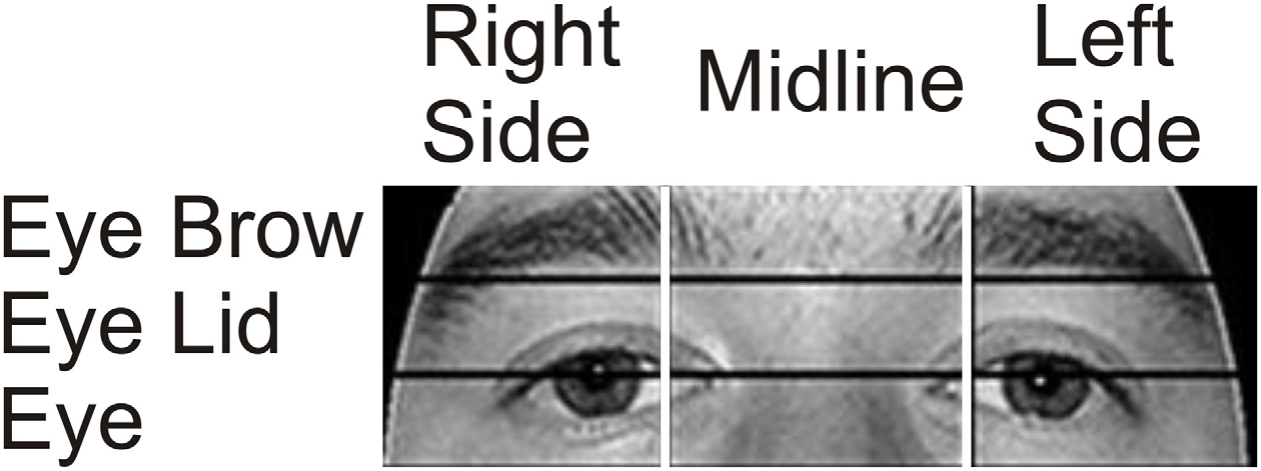
**Eye area ROIs**. The area around the eyes was further divided into two sets of three smaller ROIs to examine in more detail which areas received the most focus. In one analysis, the x-axis was divided into three vertical ROIs containing (from left to right on the image) the Right Side of the Face, the Midline, and the Left Side. In a separate analysis, the region was divided into three horizontal rows containing (from top to bottom) the Eye Brow, the Eye Lid, and the Eyes.

Previous research has shown that in healthy individuals fixations are typically made to the top of the nose, and scan paths move between the eyes and the nose. In order to determine whether our participants targeted similar facial locations, we re-examined fixations in the eye region using two sets of three smaller ROIs. In one analysis, the x-axis of the image was divided into three columns resulting in a Right Side, Midline, and Left Side ROI. In a separate analysis, the same eye region was divided into three horizontal rows resulting in Eye Brow, Eye Lid, and Eye ROIs (Figure 3).

### Experiment two—face perception in a restricted-view task

#### Participants

The entire PCA group (RB, SS, AP, MTB, and PLH) was recruited for this experiment, along with the same undergraduate control participants. Three of the four age-matched healthy controls from Experiment 1 also participated in Experiment 2 (3 females, all right handed, Mean age = 73 years). The fourth age-matched control voluntarily withdrew from the study following completion of Experiment 1; therefore one new age-matched control was recruited for Experiment 2.

#### Procedure

Prior to the start of the experiment written and verbal instructions were provided followed by a demonstration of the computer based tasked. Participants were seated at a desk or table ~50 cm away from a 15" laptop with touch-sensitive computer screen on which the “Viewing Window” task was presented. The Viewing Window is a computer based task developed in-house, written in Matlab® (Mathworks, Natick, MA). This task was initially developed in our laboratory to investigate visuomotor adaptation during an object identification task (Baugh and Marotta, 2007, 2009; Baugh et al., 2011; Lawrence-Dewar et al., 2012).

For this variation of the previous task, a single face was presented at the center of the screen with a Gaussian blur applied so that features of the image were not distinguishable. However, some information regarding the overall location and dimension of the face was attainable. In order to view part of the image in perfect clarity, a small user controlled “Viewing Window” (1.3 cm in diameter) could be moved around the touch sensitive screen to explore the image. However, the focus-window did not appear until the participant touched the stylus to the screen in order to avoid positional biases. Participants were instructed to touch the stylus to the computer screen as soon as the blurred face was visible, and to explore the underlying image with the focus-window. The faces presented in Experiment 2 were a separate set of stimuli, from the same database, that was prepared in the same way as Experiment 1. The remainder of the procedure was identical to Experiment 1, except that the PCA patients gave verbal responses that were entered by the experimenter.

#### Data analysis

Behavioral measures examined included the number of errors and the scanning pattern revealed by the location and path of the Viewing Window. The same ROIs defined in Experiment 1 were used in Experiment 2. Statistical analyses were performed in the same manner as Experiment 1.

## Results

### Experiment 1—face perception in an open-view task

#### Aged vs. young controls

**Figure 4:**
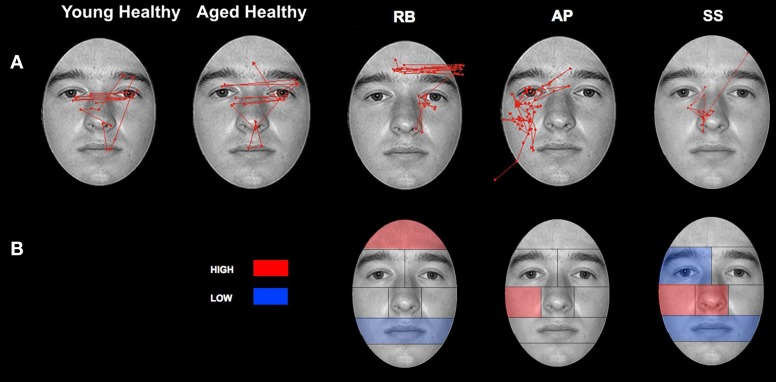
**Patterns of scanning in healthy controls and PCA Patients during the open-view face matching task. (A)** Representative scan patterns observed in young and healthy controls and PCA patient RB, AP, and SS. **(B)** Face ROIs in which PCA patients RB, AP, and SS attend to more (red) and less (blue) than age-matched controls as measured by proportion of fixation duration.

**Figure 5:**
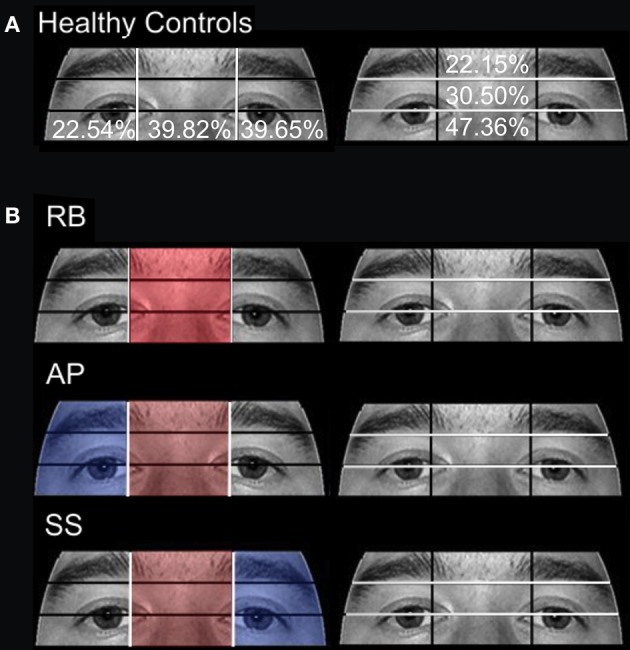
**Attention to eye region ROIs during the open-view face matching watch. (A)** The mean percentage of duration observed in Aged healthy controls is overlaid on each ROI. **(B)** The proportion of fixation durations of PCA patients RB, AP, and SS were compared to the means of Aged healthy controls with red indicating areas of greater duration and blue indicating areas of less duration. The White lines indicate the boundaries of the ROIs.

Comparisons between control groups were conducted using a two-tailed independent-samples *t*-test. The results showed no significant differences in errors when aged (*M* = 83.04%, *SD* = 17.59) and young (*M* = 86.31%, *SD* = 12.82) controls were compared [*t*_(14)_ = 0.748, *p* > 0.05]. Gaze was directed primarily to central ROIs (see Figure 4A for representative scan paths), with the highest proportion of fixation duration occurring within the Eyes, Nose, and Mouth ROIs. Scan paths of young and aged healthy individuals were strongly correlated *r* = 0.95, *n* = 8, *p* < 0.01. A closer analysis of the data confirmed these patterns. Due to the overall lack of significant differences found between aged and young controls, further discussion and comparison to individuals in PCA group will be limited to the aged control group. Examination of the smaller ROIs around the eye region revealed that aged controls spent the greatest amount of time viewing the Vertical Midline and Left Side, as well as the Eye Lid and Eye ROIs (Figure 5A).

#### Patients

A signal detection analysis was conducted on the performance of the aged control and patient groups to distinguish performance on the same/different face matching task independent of response bias. Single sample *t*-tests failed to confirm that *d*' scores were different from zero for either the aged controls [*d*′ = 2.95, *SEM* = 1.21, *t*_(3)_ = 2.43, *p* = 0.093], or patients [*d*′ = 0.41, *SEM* = 0.74, *t*_(2)_ = 0.55, *p* = 0.64]. The result was surprising given a *d*′ equal to 2.95 suggesting a lack of statistical power rather than an absence of discrimination. An independent samples *t*-test also failed to confirm that the aged controls outperformed the patients, *t*_(5)_ = 1.68, *p* = 0.14. But, once again, the large difference between a mean *d*′ of 2.95 and a mean *d*′ of 0.41 is more an indication of statistical complications due to the small sample size as a result of the inability of some of the PCA patients to calibrate on the eye-tracker. An analysis of response bias failed to confirm that either aged controls (*C* = −0.05, *SEM* = 0.21) or patients (*C* = 0.72, *SEM* = 0.48) were biased to respond same or different, relative to chance, all *ps* > 0.25. Although statistically uncorroborated, it does appear that aged controls outperformed the patients and that difference was independent of response bias.

***Patient RB***. Compared to healthy age-matched participants (95% CI [55.05%, 100%]), RB made significantly more errors in judging the two faces as being the same or different, making a correct judgment on only 35.71% of trials. Additionally, RB's scanning pattern differed from controls in that she spent longer looking at the Forehead ROI (22.37%; Controls' 95% CI [0.5%, 13.56%]) and less time looking at the Mouth ROI (4.6%; Controls' 95% CI [5.59%, 27.79.56%]) (Figures 4A,B). Parsing of the eye ROIs revealed that RB produced a longer proportion of fixation durations in the Midline (46.43%) compared to healthy controls (95% CI [34.84%, 44.79%]) (Figure 5B).

***Patient SS***. SS made significantly more errors than aged controls, making a correct matching judgment on 53.57% of trials. SS's scanning pattern deviated from controls in that he spent longer looking at the Nose (57.89%; Controls' 95% CI [9.64%, 26.27%]) and Right Cheek (2.56%; Controls' 95% CI [0%, 1.95%]) ROIs, but less time looking at the Right Eye (17.98%; Controls' 95% CI [18.29%, 32.19%]) and Mouth ROIs (4.26%) (Figures 4A,B). Parsing the eye ROIs revealed that compared to aged controls, SS showed significantly longer proportion of fixation duration to the Midline (59.26%), and significantly shorter proportion of fixation duration to the Left Side (16.3%; Controls' 95% CI [25.14%, 50.15%]) (Figure 5B). This central focus by SS is most likely the result of his self-reported strategy of focusing on the nose for face matching purposes.

***Patient AP***. AP did not make more errors than her healthy counterparts, as she made correct judgments on 60.71% of trials. When viewing the face stimuli, AP's scan pattern revealed that she spent a longer time viewing the Right Cheek (3.47%) ROI than controls (Figures 4A,B). Parsing the eye ROIs, we found that AP produced a significantly longer proportion of fixation durations to the Midline (53.81%), and shorter proportion of fixation durations to the Right Side ROI (10.12%; Controls' 95% CI [11.01%, 34.08%]) compared to controls (Figure 5B). AP's increased focus on the right cheek may partially be due to the high contrast border between the edge of cheek and the black background.

### Experiment 2—face perception in a restricted-view task

#### Aged vs. young controls

**Figure 6:**
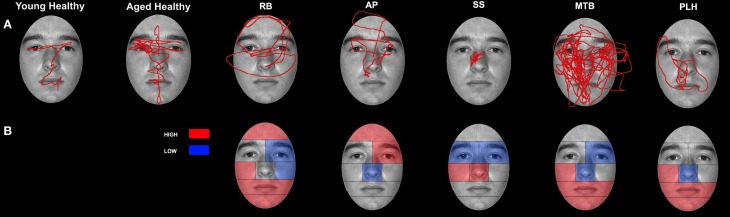
**Patterns of scanning in healthy controls and PCA Patients during the restricted-view face matching task. (A)** Representative scan paths observed in young and healthy controls and all PCA patients. **(B)** Face ROIs in which PCA patients attend to more (red) or less (blue) than age-matched controls as measured by proportion of fixation duration.

**Figure 7:**
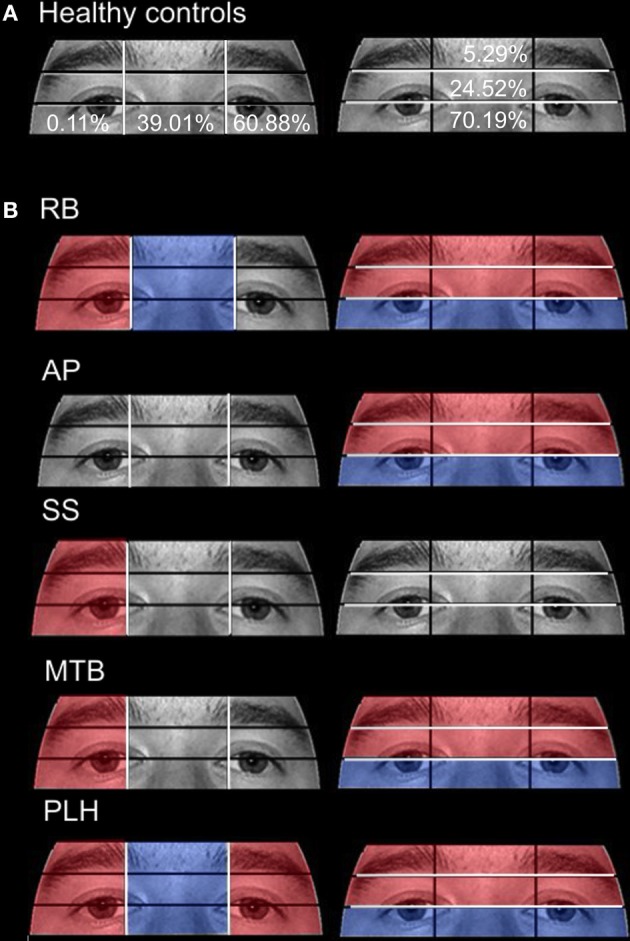
**Attention to eye region ROIs during the restricted-view face matching task. (A)** The mean percentage of duration observed in Aged healthy controls is overlaid on each ROI. **(B)** The proportion of fixation durations of PCA patients were compared to the means of Aged healthy controls with red indicating areas of greater duration and blue indicating areas of less duration. The white lines indicate the boundaries of the ROIs.

There were no significant differences in accuracy found when aged (*M* = 85.38%, *SD* = 7.76) and young (*M* = 88.69%, *SD* = 10.42) controls were compared [*t*_(14)_ = 0.246, *p* > 0.05]. While exploring the face stimuli with the Viewing Window, control participants spent the largest proportion of their viewing time examining the Left Eye, Nose, and Left Cheek ROIs. Scan paths mainly centered on the T zone containing the eyes, nose, and mouth (see Figure 6A for representative scan paths). The Viewing Window paths generated by the young and aged healthy individuals were strongly correlated *r* = 0.91, *n* = 8, *p* < 0.01. A closer analysis of the data confirmed these patterns. Due to the overall lack of significant differences found between aged and young controls, further discussion, and comparison to individuals in PCA group will be limited to the aged control group. Examination of the smaller ROIs around the eye region revealed that aged controls spent the most time viewing the Vertical Left Side as well as the Eye ROIs (see Figure 7A).

#### Patients

A signal detection analysis was conducted on the performance of the aged control and patient groups to distinguish performance on the same/different face matching task independent of response bias. An independent samples *t*-test confirmed that the aged controls (*d*′ = 2.87, *SEM* = 0.74) outperformed the patients (*d*′ = −0.12, *SEM* = 0.26) in the same/different Viewing-Window test, *t*_(7)_ = 2.94, *p* < 0.05. Additional single sample *t*-tests comparing *d*′ against zero confirmed that whereas the aged controls discriminated same from different faces, *t*_(3)_ = 3.87, *p* < 0.05, the patients did not, *t*_(4)_ = 0.45, *p* = 0.68. An analysis of response bias failed to confirm that either aged controls (*C* = −0.15, *SEM* = 0.31) or patients (*C* = 0.30, *SEM* = 0.13) were biased to respond same or different, relative to chance, all *ps* > 0.25. In conclusion, aged controls outperformed the patients and that difference was independent of response bias.

***Patient RB***. RB made significantly more errors than controls (95% CI [73.03%, 97.73%]) in making face comparisons in Experiment 2, correctly judging the face stimuli as the same or different in only 39.29% of trials. Compared to age-matched control participants, RB produced a significantly longer proportion of the viewing duration in the Mouth (21.73%; Controls' 95% CI [1.21%, 11.92%]), Chin (6.72%; Controls' 95% CI [0%, 3.78%]), Forehead (5.75%; Controls' 95% CI [0%, 5.27%]), and Right Cheek (3.29%; Controls' 95% CI [0%, 0.15%]) ROIs, with a significantly shorter proportion of the viewing duration in the Left Eye (15.14%; Controls' 95% CI [18.78%, 34.35%]) and Left Cheek (14.96%; Controls' 95% CI [15.61%, 42.98%]) regions (Figures 6A,B). In the eye-region ROIs, RB produced a significantly higher proportion of viewing duration in the Right Side (0.81%; Controls' 95% CI [0%, 0.31%]), the Eye Brow (18.71%; Controls' 95% CI [0%, 11.69%]), and Eye Lid (35.80%; Controls' 95% CI [17.71%, 31.33%]) ROIs. At the same time, RB produced a significantly shorter proportion of viewing duration in the Midline (32.68%; Controls' 95% CI [33.34%, 44.68%]) and Eye (45.5%; Controls' 95% CI [57.83%, 82.55%]) ROIs (Figure 7B).

***Patient SS***. SS made significantly more errors in matching the two faces, making correct judgments on 57.14% trials. Compared to the age-matched control group, SS produced significantly longer proportion of viewing duration in the Nose (63.49%; Controls' 95% CI [27.66%, 35.90%]) and Right Cheek (0.94%) ROIs, but shorter proportion of viewing duration in the Left Eye (11.92%) and Right Eye (0.59%; Controls' 95% CI [0.82%, 4.64%]) regions (Figures 6A,B). Parsing the eye ROIs, we found that SS showed significantly longer proportion of viewing duration in the Right Side (1.23%) compared to aged controls (Figure 7B).

***Patient AP***. AP made significantly more errors judging the similarity of faces than controls, making a correct match on only 35.71% of trials. AP's scanning pattern showed a significantly longer proportion of viewing duration in the Left Eye (40.75%) and Forehead (12.78%) ROIs, with significantly lower proportion of viewing duration in the Nose (17%) region (Figures 6A,B). Parsing the eye ROIs revealed that AP produced significantly longer proportion of viewing duration in the Eye Brow region (31.32%) and Eye Lid region (33.69%) compared to controls, while her proportion of viewing duration was significantly shorter in Eye Region (35%) (Figure 7B). Overall, AP's pattern of behavior in the RV task suggests a focus on areas of high contrast (e.g., the eyebrow and the border of the forehead and black background).

***Patient MTB***. MTB made significantly more errors than the healthy age-matched control group, making correct judgments on 55.56% of trials. Compared to controls, MTB showed a significantly longer proportion of viewing duration in the Mouth (28%), Chin (14.99%), and Right Cheek (2.11%) ROIs, with a significantly shorter proportion of viewing duration in the Left Eye (16.56%) and Nose (15.52%) regions (Figures 6A,B). Within the parsed eye ROIs, MTB produced significantly higher proportion of viewing duration in the Right Side (3.77%), Eye Brow (17.55%), and Eye Lid (35.47%) ROIs, and produced significantly shorter proportion of viewing duration in Eye ROI (46.98%) (Figure 7B). Given her preference for viewing the Eye Brow and Eye Lid Regions, it seems that, like AP, MTB appears to be focusing on high-contrast areas like the eyebrows rather than focusing on the eyes themselves, as the controls did.

***Patient PLH***. PLH produced significantly more errors, making correct judgments on only 42.86% of trials. Compared to the age-matched controls, PLH showed significantly longer proportion of viewing duration in the Mouth (21.61%), Chin (3.84%), and Right Cheek (0.23%) ROIs, with significantly shorter proportion of viewing duration in the Nose (12.22%) region (Figure 6B). Parsing the eye ROIs revealed that PLH showed significantly longer proportion of viewing duration in the Right Side (0.93%), Left Side (73.9%), Eye Brow (23.16%), and Eye Lid (34.99%), and showed significantly shorter proportion of viewing duration for Midline (25.17%) and Eye (41.86%) (Figure 7B). This pattern suggests that PLH spent more time viewing areas of high contrast, such as the eyebrows, rather that on areas like the eyes themselves.

An overview of the results for both experiments is presented in Table 2.

**Table 2:** **Summary table for the PCA patients' and aged controls' accuracy results and the percent duration spent in each of the major ROIs for Experiment 1 [Eye tracking (ET)] and Experiment 2 [Viewing Window (VW)]**.

	**Accuracy (%)**		**Forehead (%)**		**R Eye (%)**		**L Eye (%)**		**R Cheek (%)**		**Nose (%)**		**L Cheek (%)**		**Mouth (%)**		**Chin (%)**	
	**ET**	**VW**	**ET**	**VW**	**ET**	**VW**	**ET**	**VW**	**ET**	**VW**	**ET**	**VW**	**ET**	**VW**	**ET**	**VW**	**ET**	**VW**
RB	**35.71**	**39.29**	**22.37**	**5.75**	28.57	2.69	26.06	**15.14**	0.63	**3.29**	15.62	29.75	1.78	**14.96**	**4.60**	**21.73**	0.36	**6.72**
SS	**53.37**	**57.14**	1.10	0.08	**17.98**	**0.59**	14.80	**11.92**	**2.56**	**0.94**	**57.89**	**63.49**	1.40	22.43	**4.26**	2.91	0.00	0.00
AP	60.71	**35.71**	7.79	**12.78**	18.74	3.53	32.07	**40.75**	**3.47**	0.00	23.15	**17.00**	1.02	17.48	12.44	5.99	0.00	2.48
MTB	–	**55.56**	–	1.48	–	3.06	–	**16.56**	–	**2.11**	–	**15.52**	–	18.28	–	**28.00**	–	**14.99**
PLH	–	**42.86**	–	5.26	–	2.19	–	31.34	–	**0.23**	–	**12.22**	–	23.33	–	**21.61**	–	**3.84**
Aged	83.04	85.38	7.03	1.57	25.24	2.73	28.26	26.56	0.9	0.04	17.96	31.78	1.85	29.3	16.69	6.56	2.08	1.47

Bold values indicate significant differences from aged controls.

## Discussion

The responses of individuals with PCA to basic perceptual tasks, such as object identification using line drawings, suggest that they are unable to take into account all of the available information when viewing an image. The patients persistently attempted to identify images based on very select information extracted from a small part of the overall picture. For example, during preliminary testing, RB asked if a line drawing of a beaver was a “path,” pointing to the cross-hatched pattern on the animal's tail. RB went on to identify a picture of a camera as a “tunnel,” while pointing at the camera's lens. Clearly, RB and the other individuals with PCA suffer from a severely restricted window of visual focus; they do not seem to be able to “see” the entire object. Similarly, many of the PCA patients have great difficulty with face recognition. Again, preliminary testing revealed that these individuals often hone-in on one specific aspect or feature of a face, such as hair color or eyebrow shape, in order to identify the individual. As a result of this restricted focus, it is sometimes the case that larger and more “obvious” identifying features, such as skin color, are overlooked.

In Experiment 1, healthy controls spent the longest time viewing the regions of the image that make up the central “T-shape” of facial features, which includes the eyes, the nose, and the mouth. Further analysis revealed that fixations that fell within the areas surrounding the eyes preferentially targeted the regions containing the eyes themselves (as opposed to the eyebrows), as well as the area between the eyes and the upper nose. In contrast, PCA patients, RB, AP, and SS, showed a tendency to spend more time examining “non-diagnostic” areas of the face—such as the forehead and the cheeks. These areas provide very little featural or configural information that is useful for performing a face-matching task. Despite the patients' poor performance, the results of the open-view task clearly demonstrate that individuals with PCA are not impaired in their ability to scan an entire image. For example, in the open-view task, the PCA patients often showed fixation durations to key regions of the face—such as the eyes, nose, and mouth—that were no different from controls. It seems, however, that despite their intact scanning ability, the PCA patients are unable to combine individual face elements into a cohesive and recognizable whole and attempt to identify images based on a single feature.

The scanning behavior of the PCA patients does not obviously correspond to that seen in cases of typical face blindness. For example, we did not witness a tendency for individuals with PCA to spend a disproportionate amount of time looking at the mouth—a behavior that seems to be common among individuals with prosopagnosia (Le et al., 2003; Caldara et al., 2005; Barton et al., 2006; Bukach et al., 2008; Orban De Xivry et al., 2008). Similarly, the PCA patients' behavior did not perfectly mirror that previously noted in individuals with simultanagnosia, who have been shown to spend less time looking at the eye-region and more time looking at areas of high contrast (Dalrymple et al., 2011a,b). Instead, the individuals with PCA showed a unique scan pattern, spending a similar amount of time viewing the central ROIs as controls, but with an added tendency to spend more time scanning non-central facial features. The natural scanning behavior of individuals with PCA seems far more haphazard—or random—compared to the precise and directed fixation patterns exhibited by controls.

In contrast to the open-view task in Experiment 1, during the restricted-view task in Experiment 2, aged controls showed quite lateralized scanning patterns, spending more of their time in the ROIs on the right side of the computer screen. The majority of the healthy participants were right-handed and used their right hands to manipulate the stylus that was used to control the Viewing Window. Therefore, it is possible that participants were able to extract enough featural and configural information from the left half of the face that they could avoid having to produce more effortful cross-body arm movements to inspect the right side of the face. Indeed, the control groups' accuracy scores for the face matching tasks in Experiments 1 and 2 were similar (83.04 and 85.38%, respectively), indicating that neither the Viewing Window nor the left-sided bias in the restricted-view task had a detrimental effect on healthy individual's abilities to perform the task. An alternate explanation for the rightward scanning bias that should be considered is the emphasis on parts-based, or local processing, brought about by the Viewing Window. Parts-based processing has been associated with processing systems in the left hemisphere (Lobmaier et al., 2008), and the restricted-view task is designed to limit participants to a parts-based approach because configural information cannot be acquired easily using the Viewing Window. Thus, the bias toward the right visual field could be due to an increased reliance on parts-based processing from the left hemisphere.

The PCA group, as a whole, demonstrated a pattern of behavior in the restricted-view task that was very different from that of the age-matched healthy individuals, and their movement of the Viewing Window was far less restricted to the right side of the computer screen (despite four of the five patients being right handed). Instead, the individuals with PCA showed a greater focus on peripheral face-regions, with PCA patients tending to trace the outline of the face—following the high-contrast border between the face and the black background. PCA patients also spent a disproportionate amount of time viewing the upper regions of the eye-ROIs, which contain the “high-contrast” eyebrows rather than the eyes. This behavior is very similar to that which has previously been associated with simultanagnosia; Dalrymple and colleagues showed that individuals with simultanagnosia spend more time looking at areas of high contrast when compared to healthy control subjects (Dalrymple et al., 2011a,b). This similarity, combined with the previous observation that the face-viewing behavior of PCA patients does not directly resemble that of individuals with prosopagnosia, suggests that it is the reduced effective field of vision exhibited by PCA patients that is playing a significant role in their face perception deficits.

As a group, the PCA patients showed a number of definitive differences compared to the controls. However, each individual also displayed quite a unique pattern of behavior that often differed from others within the PCA group. There was no indication of a stark difference in these patterns associated with accuracy on a particular trial. PCA is the result of a progressive cortical degeneration that can produce distinct patterns of symptoms depending on the precise cortical areas affected by the disease. As such, the behaviors demonstrated in the current study are likely the result of numerous independent, yet often related disorders, such as prosopagnosia, simultanagnosia, visual neglect, optic ataxia, gaze apraxia, etc. These differences between individuals are often subtle, but they can have a large impact on studies such as this one. For example, individuals who suffer to a greater extent with visuomotor or motor-guidance symptoms may have far more of an issue with the restricted-view task, due to the required use of a stylus. An examination of motor behavior in a grasping task in the same PCA patients presented here is described in an accompanying paper (see Meek et al., 2013).

## Concluding remarks

When designing these experiments, we wondered whether the Viewing Window–a restricted-view task–would improve the performance of PCA patients in a face-matching task. Just as a restricted-view task can aid reading by allowing a patient to process one letter at a time, we hypothesized that the Viewing Window could aid in face perception. Perhaps knowing that they were only seeing a small part of the face at any one time would encourage the individuals with PCA to more fully explore parts of the face. Instead, it appears that the perceptual deficits present in most of our PCA group prevented them from even knowing what “part” of the face they were looking at through the focus-window. However, an advantage of the Viewing Window task did make itself clear—no need for calibration. Two members of our PCA group were only able to participate in the Viewing Window task because they were unable to maintain fixation long enough to complete calibration for the eye-tracker. Even the age-matched healthy control group was often difficult to calibrate on the eye-tracker because of prescription glasses and rapid fatigue.

Previous research, recording eye movements during manually operated focus-window tasks, like the Viewing Window task, has found that gaze patterns closely match the paths of the focus-windows (Baugh and Marotta, 2007; Dalrymple et al., 2007; James et al., 2010). The highly portable nature of the task, combined with compatibility with prescription eyeglasses, better patient comfort relative to head mounted eye-tracking systems, and the advantage of not having to calibrate participants, suggests an important possible role for the Viewing Window task in assessing the visuomotor performance of elderly or patient groups with neurological injury (Lawrence-Dewar et al., 2012).

Individuals with PCA produced unique scan paths that often focused on non-diagnostically useful areas of the face, rather than specific facial features. Attention to these locations was not altered by the application of a restricted viewing aperture, suggesting difficulty in face processing at either the configural or featural level. These results suggest that a reduced effective field of vision, along with basic perceptual impairments, play a major role in the face-perception deficits observed in PCA. In fact, it appears that the deficit in PCA patients is so severe that it results in an extreme dependence on local processing, such that even a feature-based approach is not possible.

### Conflict of interest statement

The authors declare that the research was conducted in the absence of any commercial or financial relationships that could be construed as a potential conflict of interest.
